# A requirement of serotonergic p38α mitogen-activated protein kinase for peripheral immune system activation of CNS serotonin uptake and serotonin-linked behaviors

**DOI:** 10.1038/tp.2015.168

**Published:** 2015-11-03

**Authors:** N L Baganz, K M Lindler, C B Zhu, J T Smith, M J Robson, H Iwamoto, E S Deneris, W A Hewlett, R D Blakely

**Affiliations:** 1 Department of Pharmacology, Vanderbilt University School of Medicine, Nashville, TN, USA; 2 Osher Center for Integrative Medicine, Vanderbilt University School of Medicine, Nashville, TN, USA; 3 Department of Neuroscience, Case Western Reserve University, Cleveland, OH, USA; 4 Institute for Psychiatric Neuroscience, Nashville, TN, USA; 5 Department of Psychiatry, Vanderbilt University School of Medicine, Nashville, TN, USA

## Abstract

Alterations in central serotonin (5-hydroxytryptamine, 5-HT) neurotransmission and peripheral immune activation have been linked to multiple neuropsychiatric disorders, including depression, schizophrenia and autism. The antidepressant-sensitive 5-HT transporter (SERT, *SLC6A4*), a critical determinant of synaptic 5-HT inactivation, can be regulated by pro-inflammatory cytokine signaling. Systemic innate immune system activation via intraperitoneal lipopolysaccharide (LPS) injection rapidly elevates brain SERT activity and 5-HT clearance. Moreover, the pro-inflammatory cytokine interleukin (IL)-1β rapidly stimulates SERT activity in raphe nerve terminal preparations *ex vivo*, effects that are attenuated by pharmacological p38 MAPK inhibition. To establish a role of serotonergic p38α MAPK signaling in LPS/IL-1β-induced SERT regulation and attendant behavioral responses, we pursued studies in mice that afford conditional elimination of p38α MAPK in 5-HT neurons (p38α^5HT−^). We found p38α^5HT−^ and control (p38α^5HT+^) littermates to be indistinguishable in viability and growth and to express equivalent levels of SERT protein and synaptosomal 5-HT transport activity. Consistent with pharmacological studies, however, IL-1β fails to increase SERT activity in midbrain synaptosomes prepared from p38α^5HT−^ animals. Moreover, although LPS elevated plasma corticosterone and central/peripheral pro-inflammatory cytokines in p38α^5HT−^ animals, elevations in midbrain SERT activity were absent nor were changes in depressive and anxiety-like behaviors observed. Our studies support an obligate role of p38α MAPK signaling in 5-HT neurons for the translation of immune activation to SERT regulation and 5-HT-modulated behaviors.

## Introduction

Depression remains the leading cause of disability worldwide.^1^ Although the etiology of depression and other mood disorders is complex, multiple studies have reported that depressed subjects display an elevation of pro-inflammatory cytokines (see Raison *et al.*^2^ for review). Experimental and/or therapeutic immune system manipulations, as with administration of cytokines or viral/bacterial mimetics such as poly I:C and lipopolysaccharide (LPS), result in mood alterations in humans.^3^ We^4^ and others^2, 3, 5^ have hypothesized that inappropriate activation of immune signaling mechanisms may also contribute to risk for mood disorders in the absence of environmental triggers. Significant evidence points to a bidirectional interaction between the immune system and serotonin (5-hydroxytryptamine, 5-HT) signaling in both the brain and periphery.^2, 6^ Peripheral immune system stimulation and/or inflammatory cytokines have been found to modulate 5-HT neuron activation, 5-HT synthesis and 5-HT release, and alter levels and/or signaling of various 5-HT receptor subtypes.^7, 8, 9, 10, 11, 12, 13, 14, 15, 16^

We have provided evidence using cell and animal models that local and systemic immune modulation can influence the antidepressant-sensitive 5-HT transporter (SERT).^4^ SERT proteins are critical for efficient clearance of the neurotransmitter after release, and represent the most common target for the pharmacological treatment of mood disorders. Using cells derived from rodent mast cells (RBL-2H3) or 5-HT neurons (RN46A), as well as transfected cells, we demonstrated a role for p38 MAPK in the regulation of SERT, with evidence supportive of rapid (minutes), trafficking-independent effects.^17, 18, 19^ Subsequently, we demonstrated the engagement of p38 MAPK signaling in the stimulatory actions of interleukin (IL)-1β and tumor necrosis factor (TNF)-α on SERT, findings gathered both in the RN46A model and mouse brain synaptosomes.^20^ Most remarkably, we found that peripheral activation of the innate immune system with LPS leads to a rapid (1 h) stimulation of central nervous system (CNS) SERT activity, accompanied by an acceleration of 5-HT clearance rate and alterations in SERT-dependent behaviors.^4^ These effects were lost in mice treated with the p38 MAPK inhibitor SB203580, and were absent in interleukin-1 receptor type I (IL-1R) knockout (KO) mice.^4^ Although these efforts drew attention to SERT as a mediator of behavioral changes linked to peripheral immune activation, our reliance on pharmacological methods and constitutive KO models left unsettled the sites of IL-1R/p38 MAPK expression involved. Peripheral LPS increases CNS IL-1β at a time coinciding with SERT upregulation.^21^ In addition, the stimulatory effects of systemic LPS on SERT are blocked by *in vitro* incubation of synaptosomes with a p38 MAPK inhibitor,^4^ providing evidence that p38 MAPK signaling within serotonergic terminals, downstream of presynaptic IL-1Rs, translates immune activation to changes in SERT and SERT-modulated behaviors.

In the current report, we describe studies examining the impact of systemic LPS in mice exhibiting a selective elimination of p38α MAPK in 5-HT neurons. We find that these mice fail to translate acute peripheral LPS injections into increased CNS SERT activity, despite normal peripheral stress responses and CNS cytokine induction. Moreover, raphe p38α MAPK excision resulted in behavioral resilience to acute LPS administration, supporting p38α MAPK-modulated 5-HT signaling as a key determinant in the behavioral manifestations of innate immune activation.

## Materials and methods

### Animals

Mouse experiments were performed under a protocol approved by the Vanderbilt Institutional Animal Care and Use Committee. Male animals of 8–12 weeks of age were housed on a 12:12 light cycle with food/water *ad libitum*, and were tested during the light period. Constitutive p38α KO mice are not viable.^22^ We therefore pursued two conditional strategies to selectively eliminate p38α MAPK within 5-HT neurons (p38α MAPK^5HT−^), both involving crosses to a p38α MAPK^*loxP/loxP*^ line^23, 24^ that was maintained on a C57Bl/6J background. In one case, we crossed these mice to ePet::Cre, mixed C57Bl/6J;129 background,^25^ to afford constitutive, 5-HT neuron-specific deletion of p38α MAPK (p38α^5HT−^) or controls lacking Cre expression (p38α^5HT+^). To afford p38α MAPK excision in adult animals, we also crossed p38α MAPK^*loxP/loxP*^ females to p38α MAPK^*loxP/loxP*^ males that were transgenic for a BAC bearing an estrogen receptor (ER)-Cre fusion, inserted into the *Slc6a4* (SERT, C57BL/6 background) gene locus.^26^ With the resulting progeny, we administered tamoxifen (20 mg ml^−1^; p38α^ER5HT−^) or corn oil (p38α^ER5HT+^) intraperitoneally (i.p.) for 5 consecutive days and performed biochemical assays 4 weeks later. This time period was chosen to allow for effective gene excision and elimination of kinase produced before tamoxifen injections, based on previous studies.^27^ As we detected elevations in serum corticosterone (CORT) in corn oil-injected animals, we evaluated brain 5-HT uptake regulation following LPS administration, but did not pursue behavioral studies in this model. To induce innate immune system activation, we administered LPS (i.p. 0.2 mg kg^−1^, 026:B6, ⩾10 000 eu mg^−1^ Sigma, St Louis, MO, USA, cat#L8274) or saline, followed 1 h later by killing by rapid decapitation, unless otherwise noted. The dose of LPS used was chosen to achieve a dose lower than that typically utilized for sickness models, and has been shown not to produce changes in locomotion in the open field assay.^4^

### Immunohistochemistry

Mice were anesthetized with Nembutal (70 mg kg^−1^) and intracardially perfused with 4% paraformaldehyde. Brains were then harvested and maintained in 4% paraformaldehyde overnight at 4 °C. The following day, brains were placed in 10 ml of 30% sucrose overnight. Brains were sectioned (40 μm, Leica SM 200 R, Leica Biosystems, Nussloch, Germany) and stored at −20 °C in freezing medium (30% ethylene glycol, 25% glycerol in phosphate-buffered saline (137 mM NaCl, 2.7 mM KCl, 10 mM Na_2_HPO_4_, 1.8 mM KH_2_PO_4,_ pH 7.4)) before analysis. Sections were stained free-floating with primary antibodies (rabbit P-p38 MAPK, 1:200 dilution, Cell Signaling Technologies, Danvers, MA, USA, #9211) or goat anti-5-HT, 1:1000 dilution, ImmunoStar, Hudson, WI, USA, #20079) overnight at 4 °C, and then with secondary antibodies (donkey anti-rabbit, 1:2000, Jackson Immunoresearch Laboratories, West Grove, PA, USA, cat#711-485-152, or donkey anti-goat, 1:200, Jackson Immunoresearch Laboratories, cat#705-025-00) for 1 h at room temperature. Antibody labeling was visualized on a Zeiss Axio Imager M2 (Thornwood, NY, USA) in the VUMC Cell Imaging Shared Resource (supported by NIH grants CA68485, DK20593, DK58404, DK59637 and EY08126).

### Neurotransmitter and mRNA assays

Brain samples obtained following rapid decapitation were assayed in the Vanderbilt Brain Institute Neurochemistry Core for biogenic amines, including 5-HT and metabolites, using high-performance liquid chromatography-based methods previously published by the Blakely laboratory.^28^ CORT levels were assayed from trunk blood using an ELISA kit (Enzo Life Sciences, Farmingdale, NY, USA; cat#ADI-900-097) in the Vanderbilt Conte Center Bioanalytical Core. For mRNA analyses, dissected midbrain and spleen samples were flash-frozen using liquid nitrogen and stored at −80 ^o^C until RNA extraction performed using Trizol reagent (Invitrogen, Grand Island, NY, USA) according to the manufacturer's instructions. Quantitative real-time PCR (qRT-PCR) was conducted using a KAPA SYBR-FAST qRT-PCR One-Step Kit (KAPA Biosystems, Wilmington, MA, USA). Thermocycling conditions were as follows: 42 °C for 5 min for complementary DNA synthesis, followed by 95 °C for 5 min for denaturation. Samples were then subjected to 40 cycles of 95 °C for 3 s, followed by 30-s extension at 60 °C (Eco qRT-PCR machine, Illumina, San Diego, CA, USA). Oligonucleotide primer sequences are available on request. mRNA levels were quantified from real-time PCR curves using the ΔΔ*C*_t_ method^29^ normalized to *Gapdh* expression.

### Western blot and synaptosome 5-HT uptake analyses

To quantify SERT protein levels, mice were killed by rapid decapitation. Midbrain and frontal cortex were dissected on ice and stored at −80 °C until use. Samples were homogenized in 25 mM HEPES, 25 mM sucrose, 1.5 mM MgCl_2_, 50 mM NaCl, pH=7.2, and protease inhibitor cocktail (Sigma, cat#P8346) before SDS-PAGE and were transfered to polyvinylidene difluoride membrane (Immobilon-P, Millipore, Bedford, MA, USA). Membranes were blocked in 5% nonfat dry milk in 1 × phosphate-buffered saline-0.1% Triton at room temperature for 1 h, washed twice with 1 × phosphate-buffered saline-0.1% Triton and incubated overnight at 4 °C with SERT antibody (1:3000 dilution, guinea pig anti-SERT; Frontier, Shinko-nishi, Ishikari, Hokkaido, Japan, cat#HTT-GP-Af1400-1) followed by a 1-h incubation at 4 °C with goat anti-guinea pig antibody (1:10 000 dilution; Jackson Immunoresearch Laboratories, cat#706-001-003). Bound antibody was detected on X-ray film (Kodak, Perkin Elmer, Boston, MA, USA, cat#NEF596) using enhanced chemiluminescence reagents (Perkin Elmer, Waltham, MA, USA, #NEL104001EA) and band density from digital scans used to quantified SERT levels. [^3^H] 5-HT uptake was measured in synaptosomes prepared from the midbrain, forebrain, hippocampus and striatum as previously described.^28^ Assays were conducted in 1-ml Krebs–Ringer's HEPES assay buffer (containing 130 mM NaCl, 1.3 mM KCl, 2.2 mM CaCl_2_, 1.2 mM MgSO_4_, 1.2 mM KH_2_PO_4_, 1.8 g l^−1^ glucose, 10 mM HEPES, pH 7.4, 100 μM pargyline and 100 μM ascorbic acid). After assessment of protein levels (Bradford assay, Bio-Rad, Hercules, CA, USA), 20–30 μg synaptosomes per sample (in a total volume of 200 μl) were pre-incubated at 37 °C in a shaking water bath for 5–10 min. Modifiers were then added for 10 min, and samples were incubated with 20 nM [^3^H] 5-HT 5 min at 37 °C. Uptake was terminated by adding 1 ml ice-cold Krebs–Ringer's HEPES buffer and by filtration through GF/B Whatman filters (soaked in 0.3% polyethylenimine for 1 h before experiment). Trapped radioactivity was eluted in scintillation liquid (Ecoscint H, National Diagnositics, Charlotte, NC, USA) overnight and quantified by scintillation spectrometry. Specific counts were obtained after subtraction of counts obtained from parallel samples assayed in the presence of 10 μM paroxetine.

### Acute midbrain slice recordings

Following rapid decapitation, midbrain slices (170 μm thickness from the midbrain) were prepared in oxygenated ice-cold sucrose-substituted artificial cerebrospinal fluid using a vibratome (VT1000S, Leica Biosystems) as previously described.^30, 31^ To measure basal firing activity of neurons in the dorsal raphe nuclei, cell-attached recordings were performed in artificial cerebrospinal fluid supplemented with 400 nM phenylephrine and 30 μM tryptophan at a perfusion rate of 1 ml min^−1^ at 32 °C. The glass pipettes (4 MΩ) were filled with HEPES solution (150 mM NaCl, 10 mM HEPES, 3.5 mM KCl, 2.5 mM CaCl_2_, 1.3 mM MgCl_2_ and 10 mM D-glucose, pH 7.4) and voltage-clamped at 0 V. Putative serotonergic neurons in dorsal raphe (DR) were selected based on cell soma size, induction of firing by phenylephrine and inhibition of basal firing rate to 5-HT_1A_ receptor agonist 8OH-DPAT (1 μM). Recordings were obtained with an Axopatch 200B amplifier connected to a Digidata 1322 A (both from Molecular Devices, Sunnyvale, CA, USA) interface connected to a Windows 7-based computer equipped with the Clampex 10.2 software (Molecular Devices).

### Behavioral assays

All assays were preformed in the Vanderbilt Brain Institute Neurobehavior Core Facility (supported by NICHD Grant P30 HD15052 to the Vanderbilt Kennedy Center for Research on Human Development). All treatments and assays were performed blind to genotype. Animals were tested in the elevated plus maze (EPM) before either tail suspension test (TST) or forced swim test (FST) as the latter tests are viewed as more stressful. No randomization was used in subject assignment. Following EPM assays, mice were allowed to rest for 7 days before running either the TST or FST. *EPM:* EPM assays were performed by placing mice into a custom-built maze with four arms at right angles to each other at ~40 cm off of the ground. One pair of opposing arms of the apparatus is open and exposed to bright room light (302 lux), and the other pair contains a walled enclosure afforded dim light (162 lux). Mice were allowed to freely explore the apparatus for 5 min while being positioned in the maze. Total distance traveled and number of entries into open or closed arms of the apparatus were recorded using the AnyMaze video tracking software (San Diego Instruments, San Diego, CA, USA). *TST:* The TST was performed as described by Steru *et al.*^32^ Mice were tested 1  h after i.p. injections by securely fastening the proximal end of the tail to a flat metallic surface, suspended in a visually isolated area (40 × 40 × 40 cm white box) with movements video recorded for 6 min. Time spent immobile was recorded, with immobility hand-scored from videos as the absence of movement aside from passive swaying. *FST*: FST studies were performed by placing mice into transparent cylinders filled to approximately two-thirds with tap water maintained at approximately room temperature (23±1 °C). FST activity was hand-scored from videos for time spent immobile versus struggling. Immobility was defined as the swimming just enough to stay afloat or not moving at all.

### Graphical and statistical analyses

We used Prism 6.0 (Graphpad Software, La Jolla, CA, USA) to perform statistical analyses and graph results. Sample sizes of experiments were chosen that minimized animal usage, that resulted in comparable variation between replicates and that insured detection of effects of ⩾25% difference as statistically significant. Grubb's test was used to identify and eliminate potential outliers. Data were analyzed via one and two-way analysis of variance under an assumption of normality, assessing main effects of genotype, drug and genotype × drug interactions followed by Bonferonni *post hoc* comparisons. In all tests, *P*<0.05 was taken as statistically significant.

## Results

### Activation and conditional elimination of p38α MAPK in raphe neurons

**Figure 1:**
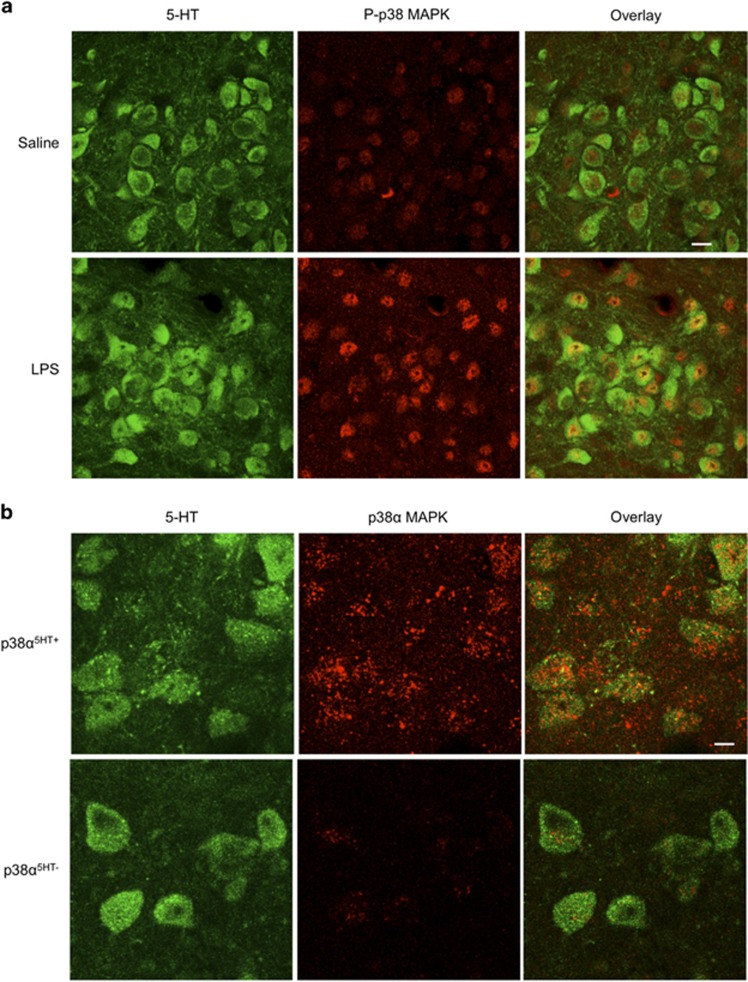
Activation and elimination of p38 MAPK in dorsal raphe (DR) 5-hydroxytryptamine (5-HT) neurons. Images of 5-HT and p38 MAPK staining for lipopolysaccharide (LPS) studies were collected from the dorsomedial division of the DR nucleus^55^ from adult male C57BL/6J mice. Images of the conditional p38α MAPK elimination experiments were obtained in the same manner. (**a**) Peripheral administration of LPS (0.2 mg kg^−1^ 1 h before being killed) elevates levels of phospho-p38 MAPK immunoreactivity in 5-HT-labeled neurons of the adult mouse dorsomedial division of the DR. Scale bar, 10 μm. (**b**) Conditional elimination of p38α MAPK immunoreactivity. Immunofluorescence for p38α MAPK is presented for floxed p38α MAPK mice without (p38α^5HT−^) or with (p38α^5HT+^) expression Cre recombinase in 5-HT neurons via ePET::Cre as described in Materials and Methods. Scale bar, 5 μm.

To determine whether peripheral LPS administration activates p38 MAPK specifically within 5-HT neurons of the DR, we used immunohistochemistry to label phospho-p38 MAPK (P-p38 MAPK), the activated form of p38 MAPK, in conjunction with a 5-HT antibody to co-label DR 5-HT neurons. The P-p38 MAPK antibody we used does not discriminate among p38 MAPK isoforms; however, as noted earlier, prior pharmacological, viral and short interfering RNA manipulations support an involvement of p38α MAPK in SERT regulation.^17, 18, 20, 33, 34^ Figure 1a demonstrates a low level of activated P-p38 MAPK in DR 5-HT cell bodies and surrounding neuropil 1 h post i.p. saline injections. Following LPS administration (0.2 mg kg^−1^, 1 h), a noticeable enhancement in P-p38 MAPK immunoreactivity was detected in 5-HT neurons. Constitutive p38α MAPK KO mice are not viable,^22^ and, regardless, the global deletion of the protein in multiple cell types would limit interpretation of results and preclude attribution of any observed effect to an effect in 5-HT neurons. Therefore, we pursued a conditional strategy, breeding p38α MAPK^*loxP/loxP*^ mice^24^ to p38α MAPK^*loxP/loxP*^;ePet::Cre,^27^ to selectively eliminate p38α MAPK within 5-HT neurons (p38α MAPK^5HT−^). Immunofluorescence imaging of p38α MAPK expression in DR-containing sections confirmed the loss of kinase expression in 5-HT neurons of p38α MAPK^5HT−^ animals compared with p38α MAPK^5HT+^ animals (Figure 1b).

### Impact of serotonergic p38α MAPK deletion on 5-HT biochemistry, physiology and SERT function

**Figure 2:**
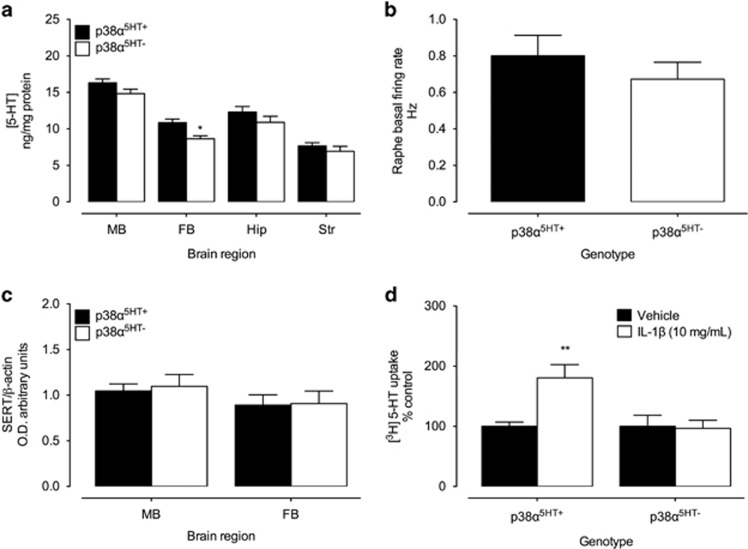
Serotonergic expression of p38α MAPK is required for acute interleukin (IL)-1β-induced SERT activation. (**a**) High-performance liquid chromatography (HPLC) analysis of 5-hydroxytryptamine (5-HT) levels in the midbrain (MB), forebrain (FB), hippocampus (Hip) and striatum (Str) from male p38α^5HT+^ and p38α^5HT−^ mice. Two-way analysis of variance (ANOVA) indicates significant region (F_3,67_=71.75, *P*<0.0001) and genotype (F_1, 67_,=4.12, *P*=0.001) effects and a nonsignificant interaction (F_3,67_=0.53, *P*=0.67). *N* for all regions=10 for p38α^5HT+^, *N*=8 for p38α^5HT−^. (**b**) Basal firing rate of dorsal raphe 5-HT neurons as assessed by cell-attached recordings of serotonergic neurons in acute midbrain slices from p38α^5HT+^ and p38α^5HT−^ mice, *N*=10 neurons from three p38α^5HT+^ mice, *N*=15 neurons from three p38α^5HT−^ mice, with data derived from two to three slices per animal. Student's two-tailed *t*-test reveals no significant genotype effect. (**c**) SERT protein expression in MB and FB of p38α^5HT+^ and p38α^5HT−^ mice assessed by western blotting. Two-way ANOVA indicates no significant region or genotype effects. *N*=7 for all groups. (**d**) Transport of [^3^H]-5HT (50 nM) in MB synaptosomes of p38α^5HT+^ and p38α^5HT−^ mice following treatment with IL-1β (10 ng ml^−1^), compared with vehicle. Two-way ANOVA indicates significant drug × genotype interaction (F_1, 20_=6.68, *P*=0.02), drug (F_1, 20_=5.62, *P*=0.02), and genotype (F_1, 20_=6.68, *P*=0.02). Bonferroni *post hoc* shows a significant effect of IL-1β in lipopolysaccharide (LPS)-treated p38α^5HT+^, but not p38α^5HT−^ mice, compared with vehicle control counterparts. *P<0.05; **P<0.01, *N*=6 for all groups.

To determine whether p38α^5HT−^ mice exhibit alterations in 5-HT signaling capacity, we assessed midbrain, forebrain, hippocampal and striatal levels of 5-HT and metabolites using high-performance liquid chromatography evaluation of tissue extracts as noted in the Materials and methods section. We detected a small, but statistically significant effect of genotype on 5-HT levels, with a modest, region-independent reduction (10–20%) seen in the p38α^5HT−^ mice (Figure 2a). No significant alterations were found in the levels of dopamine or norepinephrine or the 5-HT metabolite, 5-hydroxyindoleacetic acid (data not shown). Whole-cell recordings of DR 5-HT neurons in acute brain slices of p38α^5HT+^ and p38α^5HT−^ mice revealed no statistically significant alterations in basal firing rates (Figure 2b). p38α^5HT−^ mice also demonstrated no changes in either midbrain or forebrain SERT levels (Figure 2c) nor was basal 5-HT uptake in midbrain synaptosomes influenced (Figure 2d). These findings indicate little or no effect of loss of p38α MAPK expression on basal serotonergic measures. However, when we queried the contribution of p38α MAPK to cytokine stimulation of SERT, a different picture emerged. Thus, whereas IL-1β, as previously published,^4^ rapidly stimulated 5-HT uptake in midbrain synaptosomes of control, p38α^5HT+^ animals, no stimulation was found with synaptosomes from p38α^5HT−^ mice (Figure 2d).

### LPS elevation of central and peripheral pro-inflammatory cytokine mRNA, as well as plasma CORT, occurs independently of serotonergic p38α MAPK expression

**Figure 3:**
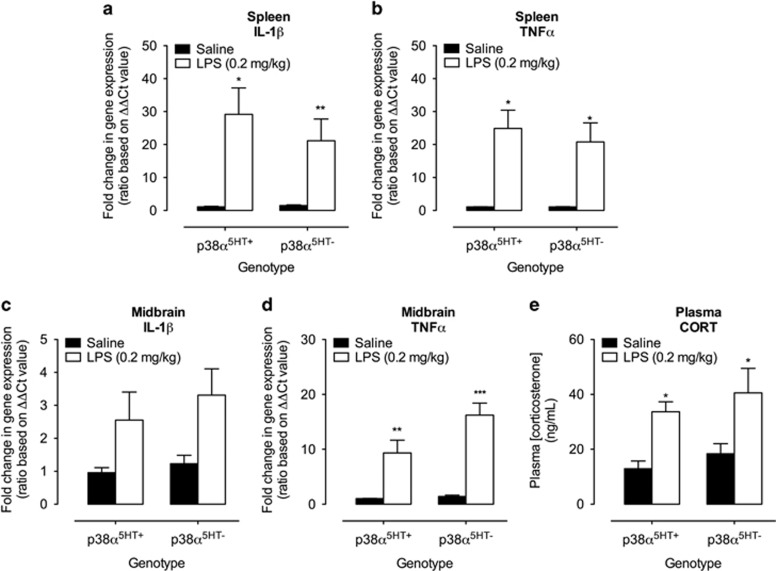
Lipopolysaccharide (LPS) induction of central and peripheral pro-inflammatory cytokines, as well as plasma corticosterone, occurs independently of serotonergic p38α MAPK expression. Quantitative PCR (qPCR) analyses of mRNA expression were assessed 1 h post saline (i.p.) or LPS (0.2 mg kg^−1^, i.p.) injection. (**a**, **b**) Two-way analysis of variance (ANOVA) demonstrates significant LPS-induced elevation of spleen interleukin (IL)-1β (F_1, 21_=20.43, *P*<0.001) and tumor necrosis factor (TNF)-α (F_1, 28_=29.20, *P*<0.001) independent of genotype (nonsignificant genotype and interaction). *N*=5–11 per group. (**c**, **d**) Two-way ANOVA demonstrates significant LPS-induced elevation of midbrain IL-1β, (F_1, 24_=8.70, *P*<0.01) and TNF-α (F_1, 21_=42.63, *P*<0.01) independent of genotype. *N*=5–8 per group. (**e**) Two-way ANOVA demonstrates significant LPS-induced elevation of serum corticosterone (CORT); drug effect F_1, 24_=15.56, *P*<0.01, independent of genotype. *N*=4–8 per group. *P<0.05; **P<0.01; ***P<0.001, Bonferroni *post hoc* tests.

Peripheral LPS administration activates the innate immune system, stimulating the release of multiple inflammatory cytokines, including IL-1β and TNF-α, in the brain and periphery,^35^ and producing a systemic stress response reflected in elevations in plasma CORT.^36^ To ensure that our conditional targeting strategy did not alter these responses, we quantified mRNA levels of IL-1β and TNF-α in periphery (spleen) and midbrain, as well as serum CORT 1 h following the administration of LPS (0.2 mg kg^−1^ i.p.) to p38α^5HT+^ mice and their p38α^5HT−^ littermates. Neither the LPS-induced increases in splenic and midbrain IL-1β and TNF-α mRNA expression nor the elevation of plasma CORT were influenced by serotonergic loss of p38α MAPK (Figure 3a–e).

### Serotonergic p38α MAPK is required for peripheral LPS stimulation of CNS SERT activity as well as LPS-induced depressive/anxiety-like behaviors

**Figure 4:**
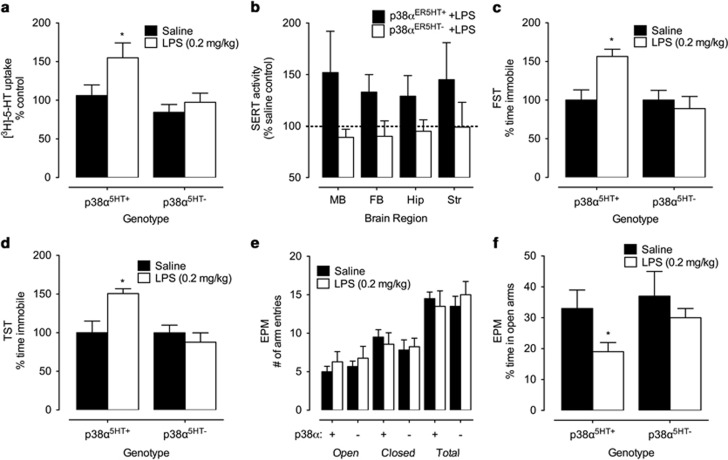
Serotonergic expression of p38α MAPK is required for lipopolysaccharide (LPS) stimulation of SERT activity and depressive/anxiety-like behaviors. (**a**) [^3^H]-5-HT (5-hydroxytryptamine) uptake (50 nM) in midbrain synaptosomes of p38α^5HT+^ and p38α^5HT−^ males 1 h post intraperitoneal (i.p.) saline or LPS (0.2 mg kg^−1^) injection. LPS increased SERT activity in midbrain synaptosomes of p38α^5HT+^, but not p38α^5HT–^, mice. Two-way analysis of variance (ANOVA) significant effect of genotype (F_1, 23_=4.31, *P*<0.05) and LPS (F_1, 23_=7.12, *P*<0.05), Bonferroni *post hoc* *P<0.05 in p38α^5HT+^ group only. *N*=6–8 per group. (**b**) Assessment of SERT activity in mice with adult excision of p38α MAPK. *Slc6a4*-ER-Cre;p38α MAPK^*loxP/loxP*^ mice were treated for 5 days with corn oil or tamoxifen as described in Methods. Four weeks later, mice were administered saline or LPS (0.2 mg kg^−1^ i.p.) 1 h before being killed. SERT activity was measured in synaptosomes prepared from midbrain (MB), forebrain (FB), hippocampus (Hip) and striatum (Str). The percentage change of 5-HT uptake activity for LPS versus saline controls is plotted for each condition. Two-way ANOVA demonstrates significant LPS effect on SERT activity in p38α^ER5HT+^ (*N*=8 for MB and FB; *N*=9 for Hip; *N*=4 for Str)-treated animals (F_3, 55_=8.56, *P*=0.005) but not in p38α^ER5HT−^ (*N*=12 for MB; *N*=10 for FB; *N*=7 for Hip; *N*=4 for Str) animals (*P<*0.05). (**c**) Percent time immobile in the forced swim test (FST) for p38α^5HT+^ and p38α^5HT-^ mice 1 h post saline or LPS (0.2 mg kg^−1^ i.p). Two-way analysis of variance (ANOVA) shows significant interaction between LPS and genotype (F_1, 15_=7.01; *P*=0.02). Immobility time was increased in p38α^5HT+^, LPS-treated mice (*P<0.05, Bonferroni *post hoc*; *N*=5 per for saline and LPS-treated animals), but not p38α^5HT-^ mice (*N*=5 for saline-treated and *N*=4 for LPS-treated animals). (**d**) Percent time immobile in the tail suspension test (TST) for p38α^5HT+^ and p38α^5HT−^ mice 1 h post saline or LPS (0.2 mg kg^−1^ i.p.). Two-way ANOVA shows significant interaction between treatment and genotype (F_1, 17_=7.83; *P*=0.01). Immobility time was increased in p38α^5HT+^ (*N*=5 per for saline and LPS-treated animals), LPS-treated mice (*P<0.05, Bonferroni *post hoc*), but not in p38α^5HT−^ mice (*N*=6 for saline-treated and *N*=5 for LPS-treated animals). (**e**) Open, closed and total number of arm entries in the EPM in p38α^5HT+^ (+) and p38α^5HT−^ (−) mice treated with saline or LPS (0.2 mg kg^−1^ i.p.). There was no significant effect of genotype or drug. (**f**) Percent time spent in the open arms of the EPM (6-min test). Two-way ANOVA shows significant effects of LPS (F_1, 17_=8.81; *P*<0.01) and genotype (F_1, 17_=4.71; *P*=0.04). LPS reduced time spent in open arms in p38α^5HT+^ (*N*=5 in saline- and LPS-treated animals) but not in p38α^5HT−^ (*N*=6 and 4 in saline- and LPS-treated animals, respectively) mice (*P<0.05, Bonferroni *post hoc*).

Observing that SERT in midbrain synaptosomes from p38α^5HT−^ mice lacked sensitivity to acute stimulation with IL-1β, we next asked whether SERT activity in these animals would also lack responsiveness to peripheral LPS administration. Consistent with previous studies of C57BL/6 mice,^4^ LPS (0.2 mg kg^−1^) stimulated [^3^H]-5-HT uptake (50 nM) in midbrain synaptosomes of p38α^5HT+^ mice, but failed to increase SERT activity in p38α^5HT−^ mice (Figure 4a). Together with our *ex vivo* IL-1β studies, these findings provide strong support for an essential role of p38α MAPK in translating acute innate immune activation to changes in SERT activity. We observed a similar inability of LPS to stimulate SERT activity when p38α MAPK excision was induced in adults using tamoxifen-treated p38α MAPK^*loxP/loxP*^ mice positive for an *Slc6a4*::ER-Cre transgene^26^ (Figure 4b).

Previously, we demonstrated that acute, 1 h systemic LPS treatment increased immobility in the FST and TST, a depressive-like effect that was not seen in SERT KO animals.^4^ In addition, treatment with the p38α MAPK inhibitor SB203580 mitigated LPS-mediated increases in immobility, although systemic drug administration precluded determination of critical sites of kinase expression. To determine whether p38α MAPK signaling in 5-HT neurons is required for LPS-mediated depressive-like behavior, p38α^5HT+^ and p38α^5HT−^ littermates were treated with LPS as in our previous report^4^ (0.2 mg kg^−1^ i.p. 1 h before testing). As expected, we observed increased immobility time in both the FST (Figure 4c) and TST (Figure 4d) in p38α^5HT+^ mice. In contrast, p38α^5HT−^ littermates failed to exhibit changes in these behaviors in response to LPS treatments. In the EPM, we detected no genotype- or treatment-related differences in the number of open, closed or total number of arm entries (Figure 4e), consistent with the dose of LPS used not influencing global locomotor activity in either p38α^5HT+^ or p38α^5HT−^ mice. However, LPS injections reduced time spent in the open arms of the maze in p38α^5HT+^ but not in p38α^5HT−^ mice (Figure 4f), consistent with an anxiety response in the former but not the latter animals.

## Discussion

That immune activation can lead to CNS-mediated physiological and behavioral changes has been clear for decades.^37, 38^ Such changes likely arise in part from a subsequent elevation of CNS pro-inflammatory cytokines, including IL-1β. Canonical signaling by the IL-1R engages a MAPK kinase signaling cascade that ultimately activates JNK and p38 MAPK signaling pathways.^39^ We found that within 1 h after peripheral immune activation with LPS, SERT activity in synaptosomes *ex vivo* and SERT-mediated 5-HT clearance *in vivo* were significantly elevated,^4^ effects abolished by pre-administration of SB203580,^4, 17, 40, 41, 42^ an inhibitor of the p38α and β MAPK isoforms.^40^ Supporting the idea that these effects were mediated through the p38α MAPK isoform, increases in SERT activity induced by anisomycin, a non-selective activator of p38 MAPKs, were suppressed using short interfering RNAs derived selectively from p38α MAPK sequences.^41^ Moreover, this stimulation led to behavioral changes known to be sensitive to acute selective serotonin reuptake inhibitor administration, suggesting that they might involve modulation of CNS 5-HT signaling pathways and be supported by modulation of SERT activity. In support of this idea, peripheral LPS administration has been found to activate c-Fos expression in raphe 5-HT neurons^43, 44^ and microdialysis studies indicate enhanced elevations in 5-hydroxyindoleacetic acid levels, consistent with elevated 5-HT uptake and metabolism, following LPS administration.^45, 46^ Finally, the behavioral significance of 5-HT neural p38α MAPK was demonstrated by Bruchas *et al.*^34^ as being critical to the translation of social defeat stress to depressive-like behavior.

We used two genetic approaches to compromise p38α MAPK signaling in 5-HT neurons. The primary approach used in this study involved elimination of p38α MAPK expression via ePet::Cre expression in animals homozygous for a floxed allele of *Mapk14*.^23, 24, 25, 27^ The ePET::Cre approach we implemented has been shown to produce efficient excision of floxed genes in the majority of CNS 5-HT neurons,^25^ with Cre expression initiating in raphe neurons by e12.5, before elaboration of serotonergic traits. Serotonergic p38α MAPK excision did not result in overt effects in morphology, physiology or behavior. Thus, we found no differences in viability, growth rates, gross physical appearance or reproduction with these animals, nor did we observe alterations in spontaneous locomotor activity, as evaluated in open field tests (data not shown). Slight changes were found in 5-HT levels across brain regions, although raphe serotonergic neuron number and size appeared grossly normal and basal firing rates recorded *ex vivo* were unchanged. In contrast, p38α MAPK excision completely eliminated the ability of peripheral LPS *in vivo* or IL-1β *ex vivo* to elevate SERT activity, supporting an essential contribution of kinase activation to SERT regulation by these inflammatory stimuli. We also induced Cre expression in adult, SERT-expressing cells via a cross of p38α MAPK^*loxP/loxP*^ animals to a line expressing Cre via an ER-SERT BAC construct.^26^ Our studies with the ER-SERT BAC line add evidence for an ongoing requirement for 5-HT neuron p38α MAPK activity in LPS-induced SERT regulation.

We^4^ and others^34^ have demonstrated that acute LPS administration generates increased immobility in both the FST and the TST. At the dose of LPS used in our tests, immobility effects in these tests are not accounted for by changes in general locomotor activity, which can be suppressed at higher doses.^4, 46, 47^ These tests have predictive utility related to antidepressant efficacy in humans, many of which target SERT.^48, 49^ Moreover, we previously found that SERT KO mice exhibit resiliency with respect to LPS-induced immobility in the TST.^4^ Our studies demonstrate for the first time a requirement for p38α MAPK expression by 5-HT neurons in LPS-induced immobility in the FST and TST. We detected no alteration in the induction of peripheral or CNS IL-1β expression following LPS administration nor were there difference in serum CORT responses, supporting the hypothesis that a p38α MAPK signaling cascade in 5-HT neurons drives LPS-induced despair behavior in the TST/FST. Finally, LPS injections have been reported to reduce time spent by mice in the open arms of the EPM,^50^ commonly inferred as an anxiety response. We observed a dependence on serotonergic p38α MAPK expression for LPS-induced reduction in time spent in the open arms, suggesting a critical contribution of altered 5-HT signaling for immune activation-triggered anxiety responses.^51, 52^

The specific target(s) of p38α MAPK within 5-HT neurons underlying the behavioral effects of LPS remain to be definitely elucidated. Here we provide correlative evidence that a plausible target as a trigger for behavioral changes is SERT. Additional studies are needed to determine whether SERT regulation through the p38α MPAK pathway has a role in biochemical and behavioral changes brought about by chronic inflammatory states. SERT is a phosphoprotein under basal conditions^53^ with evidence that a significant portion of this phosphorylation is achieved via p38 MAPK-linked pathways^18, 33^ and that basal p38 MAPK-induced SERT phosphorylation contributes to transporter surface density,^33^ whereas 5-HT affinity and transport rates are influenced by activated p38 MAPK.^41^ Studies are needed that utilize animals in which SERT is rendered insensitive to p38α MAPK modulation. Although p38α MAPK activity supports basal phosphorylation of SERT in nerve terminal preparations,^33^ specific sites that support this activation remain to be identified. A site in the cytoplasmic C terminus of human SERT (Thr616) has been proposed as a potential site for p38α MAPK regulation based on *in vitro* studies using synthetic human SERT peptides and purified p38α MAPK,^54^ although these findings are yet to be validated with the intact SERT protein, or in a cellular context. Our studies suggest that p38α MAPK-dependent SERT regulatory mechanisms may harbor risk determinants for mood disorders and targeting these pathways could provide a novel route to therapeutics, one that buffers against inappropriate SERT activation versus the current strategy of totally eliminating SERT-mediated 5-HT clearance.
